# The characteristics of the synonymous codon usage in hepatitis B virus and the effects of host on the virus in codon usage pattern

**DOI:** 10.1186/1743-422X-8-544

**Published:** 2011-12-15

**Authors:** Ming-ren Ma, Xiao-qin Ha, Hui Ling, Mei-liang Wang, Fang-xin Zhang, Shang-di Zhang, Ge Li, Wei Yan

**Affiliations:** 1 Experimental Center of Medicine, Lanzhou General Hospital, Lanzhou Military Area Command; Key lab of Stem cells and Gene Drugs of Gansu Province, Lanzhou 730000, China

**Keywords:** Hepatitis B virus, codon usage pattern, evolution, mutation pressure, translation selection

## Abstract

**Background:**

Hepatitis B virus (HBV) infection is one of the main human health problem and causes a large-scale of patients chronic infection worldwide.. As the replication of HBV depends on its host cell system, codon usage pattern for the viral gene might be susceptible to two main selections, namely mutation pressure and translation selection. In this case, a deeper investigation between HBV evolution and host adaptive response might assist control this disease.

**Result:**

Relative synonymous codon usage (RSCU) values for the whole HBV coding sequence were studied by Principal component analysis (PCA). The characteristics of the synonymous codon usage patterns, nucleotide contents and the comparison between ENC values of the whole HBV coding sequence indicated that the interaction between virus mutation pressure and host translation selection exists in the processes of HBV evolution. The synonymous codon usage pattern of HBV is a mixture of coincidence and antagonism to that of host cell. But the difference of genetic characteristic of HBV failed to be observed to its different epidemic areas or subtypes, suggesting that geographic factor is limited to influence the evolution of this virus, while genetic characteristic based on HBV genotypes could be divided into three groups, namely (i) genotyps A and E, (ii) genotype B, (iii) genotypes C, D and G.

**Conclusion:**

Codon usage patterns from PCA for identification of evolutionary trends in HBV provide an alternative approach to understand the evolution of HBV. Further more, a combined selection of mutation pressure with translation selection on codon usage might shed a light on understanding the evolutionary trends of HBV genotypes.

## Introduction

Hepatitis B virus (HBV) disease is one of the main global health problems that two billion people are infected and 350 million people undergo chronic infection as well [1]. HBV belongs to the protyotype member of the family *Hepadnaviridae*, and has a compact and circular DNA genome of about 3.2 kb in length, with four overlapping open reading frames including large S region (PreS/S), PreC/C, × and P [2,3]. Moreover, the overlapping regions on the genome are helpful to study the evolution of the virus with its point mutations, because the incidence of recombination is rare and any point mutation could effect the genetic characteristics of two overlapped genes [3]. The evolution of HBV should be interactional and constrained by the overlap of genes [4]. In some cases, the evolution of one overlapping-gene protein may evolve more rapidly as a consequce of negative selection to the other,[5]. And the overlapping genes might be subject to different selections [6]. Furthermore, independent adaptive selection for both overlapping genes has been reported [7]. One of the main features of HBV are its genetic heterogeneity [8]. There are four main subtypes, namely ayw, adw, adr and ayr [9]. According to phylogenetic analysis of the complete HBV genomic sequence, 9 genotype of HBV from genotype A to I have been determined and divided into approximately twenty-five subgenotypes [10-14]. HBV genotypes show distinct geographical distributions at the level of nucleotide different more than 8% each other [11,15,16]. It is noticed that nucleotide composition comprising of HBV coding sequence with various genetic diversities is selective rather than random, because the natural selection from host is responsible for selection of various strains shaped by mutation. In previous reports, translation selection and compositional constraints under the mutational pressure are thought to be the major factors accounting for codon usage variation among genomes in microorganisms [17-24]. In some RNA viruses, compared with natural selection, mutation pressure plays a more important role in synonymous codon usage pattern [25,26]. Although it is known that compositional constraints and translation selection are the more generally accepted mechanisms accounting for codon usage bias [27-30], other selection forces have also been proposed such as fine-tuning translation kinetics selection as well as escape of cellular antiviral responses [23,31-34]. Thus, the codon usage pattern may be important in disclosing the molecular mechanism and evolutionary process of HBV to avoid host cell response. To our knowledge, it is the first systemic study to analysis the synonymous codon usage pattern and evolutional dynamics of HBV as well as the relationship between codon usage pattern of HBV and its host.

## Result

### Synonymous coodn usage in HBV

The C% and U% were higher than A% and G%, and C_3_% and U_3_% were higher than A_3_% and G_3_% in HBV (Table 1).

**Table 1:** The overall nucleotide contents and nucleotide contents at the synonymous third position of sense codons in the whole coding sequence of HBV

**No**.	T%	C%	A%	G%	T_3_%	C_3_%	A_3_%	G_3_%
1	27.88	28.16	22.21	21.75	29.94	27.27	21.23	21.56
2	28.28	28.04	21.59	22.09	29.75	27.90	20.57	21.78
3	27.35	28.20	22.04	22.42	29.40	27.04	21.19	22.37
4	27.56	28.33	21.96	22.15	29.44	27.58	20.91	22.07
5	27.85	27.87	22.26	22.02	30.23	26.33	21.75	21.69
6	27.56	28.50	21.93	22.02	29.20	27.73	21.12	21.95
7	27.60	28.50	21.93	21.97	29.27	27.73	21.12	21.89
8	27.60	28.50	21.87	22.04	29.27	27.73	21.05	21.95
9	28.09	27.38	21.88	22.64	29.94	26.10	21.55	22.40
10	28.35	27.23	23.29	21.13	31.95	25.71	22.51	19.83
11	28.12	27.48	21.88	22.52	29.73	26.53	21.48	22.26
12	27.54	27.83	21.87	22.77	28.68	27.54	21.22	22.57
13	28.21	26.97	23.04	21.78	29.04	27.58	21.65	21.73
14	27.95	28.17	21.52	22.35	29.96	27.41	20.76	21.88
15	27.95	28.11	21.79	22.15	30.14	27.04	21.19	21.63
16	27.43	28.01	21.98	22.58	29.58	26.79	21.07	22.56
17	28.65	27.79	21.75	21.82	30.55	27.04	21.14	21.28
18	28.72	27.91	21.39	21.98	29.75	26.45	21.75	22.05
19	28.57	28.40	21.21	21.82	28.25	28.35	21.15	22.25
20	28.55	28.41	21.22	21.82	28.23	28.34	21.19	22.24
21	28.57	28.39	21.22	21.82	28.23	28.34	21.19	22.24
22	28.34	28.27	21.62	21.77	29.48	27.47	21.19	21.86
23	28.22	28.07	21.66	22.04	29.66	27.05	21.24	22.04
24	28.43	28.00	21.65	21.91	30.25	27.24	21.22	21.29
25	27.57	27.99	21.81	22.62	29.71	26.79	20.94	22.56
26	28.74	27.98	21.56	21.72	29.78	26.53	21.96	21.74
27	28.60	28.24	21.39	21.77	29.34	27.14	21.70	21.83
28	28.68	28.17	21.64	21.52	29.56	27.01	21.96	21.48
29	28.78	28.14	21.43	21.65	29.60	26.97	21.74	21.70
30	28.66	28.25	21.36	21.72	29.47	26.97	21.56	22.00
31	28.78	27.68	22.10	21.45	30.76	25.77	22.13	21.34
32	29.07	27.45	21.85	21.63	30.85	25.90	21.91	21.34
33	29.08	27.56	21.65	21.71	30.76	26.25	21.65	21.34
34	28.81	27.42	22.08	21.69	30.63	25.68	22.34	21.34
35	28.39	27.90	21.71	22.01	29.67	26.72	21.52	22.08
36	28.72	27.77	21.92	21.59	30.59	26.12	22.08	21.21
37	28.98	27.52	21.89	21.61	30.63	26.16	21.87	21.34
38	28.95	27.61	21.89	21.55	30.67	26.25	22.04	21.04
39	29.05	27.42	21.85	21.68	30.89	25.81	22.08	21.21
40	28.95	27.59	21.91	21.55	30.85	25.99	21.74	21.43
41	28.42	27.75	21.79	22.04	29.89	26.46	21.78	21.87
42	28.88	27.45	21.89	21.78	30.76	25.55	22.08	21.61
43	28.47	27.65	22.25	21.63	30.91	26.21	21.74	21.14
44	28.99	27.49	21.92	21.59	30.37	26.03	22.21	21.39
45	28.99	27.45	21.95	21.61	30.55	25.90	22.12	21.42
46	28.71	27.72	21.97	21.61	30.28	26.38	22.17	21.17
47	28.71	27.72	21.97	21.61	30.28	26.38	22.17	21.17
48	27.51	27.78	22.15	22.55	29.81	26.46	21.52	22.22
49	27.43	27.93	21.98	22.66	29.21	26.91	21.19	22.68
50	27.43	27.93	21.98	22.66	29.21	26.91	21.19	22.68
51	27.41	27.95	21.92	22.73	29.33	26.66	21.26	22.75
52	27.45	27.99	21.86	22.71	29.52	26.72	21.01	22.75
53	27.49	27.95	21.84	22.73	29.52	26.60	21.26	22.62
54	27.64	27.97	21.83	22.56	29.44	27.23	20.85	22.49
55	27.45	27.91	21.96	22.68	29.33	26.72	21.32	22.62
56	27.41	27.91	22.04	22.64	29.40	26.72	21.44	22.44
57	27.60	28.50	21.87	22.04	29.27	27.73	21.05	21.95
58	27.79	28.33	22.20	21.68	29.80	27.41	21.55	21.24

The overall nucleotide composition never affects the nucleotide contents in the third site of codon in HBV coding sequence, suggesting that composition constraints may be one of the factors in affecting the codon usage pattern of HBV. For the synonymous codon usage pattern of HBV, the over-represented synonymous codons are rare in HBV coding sequence, only including UCU for Ser, in addition, the under-represented ones contain AUA for Ile, CCC for Pro, ACC for Thr, GCC for Ala, CGU and CGG for Arg (Table 2).

**Table 2:** The relationship of the synonymous codon usage pattern between HBV and human cell

Codon/Amino acid	HBV	Human^a^
**TTT(F)**	1.06	0.87

**TTC(F)**	0.94	1.13

**TTA(L)**	0.67	0.39

**TTG(L)**	1.08	0.73

**CTT(L)**	1.11	0.73

**CTC(L)**	1.22	1.22

**CTA(L)**	0.85	0.40

**CTG(L)**	1.06	2.53

**ATT(I)**	1.27	1.04

**ATC(I)**	1.26	1.52

**ATA(I)**	0.48	0.44

**GTT(V)**	1.27	0.69

**GTC(V)**	0.91	1.00

**GTA(V)**	0.65	0.42

**GTG(V)**	1.17	1.90

**TCT(S)**	1.69	1.11

**TCC(S)**	1.48	1.39

**TCA(S)**	1.28	0.84

**TCG(S)**	0.58	0.33

**AGT(S)**	1.48	0.84

**AGC(S)**	1.01	1.50

**CCT(P)**	0.99	1.12

**CCC(P)**	0.51	1.35

**CCA(P)**	1.37	1.07

**CCG(P)**	1.38	0.46

**ACT(T)**	0.89	0.94

**ACC(T)**	0.37	1.52

**ACA(T)**	1.32	1.07

**ACG(T)**	1.24	0.46

**GCT(A)**	0.99	1.09

**GCC(A)**	0.45	1.64

**GCA(A)**	1.27	0.85

**GCG(A)**	0.73	0.42

**TAT(Y)**	1.05	0.84

**TAC(Y)**	0.95	1.16

**CAT(H)**	1.21	0.81

**CAC(H)**	0.79	1.19

**CAA(Q)**	1.08	0.51

**CAG(Q)**	0.92	1.49

**AAT(N)**	1.36	0.89

**AAC(N)**	0.64	1.11

**AAA(K)**	0.73	0.82

**AAG(K)**	1.27	1.18

**GAT(D)**	1.04	0.89

**GAC(D)**	0.96	1.11

**GAA(E)**	1.23	0.81

**GAG(E)**	0.77	1.19

**TGT(C)**	0.80	0.86

**TGC(C)**	1.06	1.14

**CGT(R)**	0.48	0.51

**CGC(R)**	0.78	1.20

**CGA(R)**	0.61	0.63

**CGG(R)**	0.37	1.20

**AGA(R)**	1.49	1.20

**AGG(R)**	1.39	1.26

**GGT(G)**	0.60	0.64

**GGC(G)**	0.81	1.40

**GGA(G)**	1.36	0.98

**GGG(G)**	1.22	0.98

^a ^the synonymous codon usage pattern of human cell was calculated based on the data of the synonymous codon usage frequencies of human cell.

The codon usage bias of HBV suggests that some synonymous codons are not chosen equally and randomly.

### Genetic relationship based on synonymous codon usage in HBV

**Figure 1:**
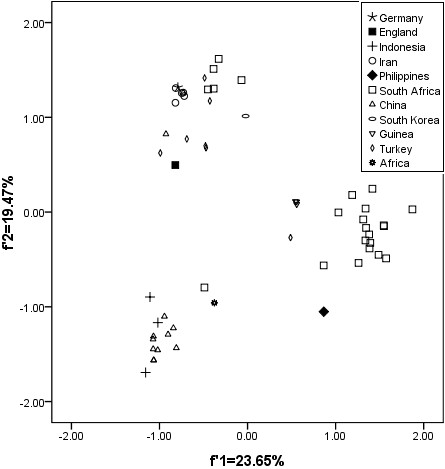
**The genetic characteristic of HBV isolated different countries**.

The PCA detected the first principal component (*f_1_'*) which can account for 23.65% of the total synonymous codon usage variation, and the second principal component (*f_2_'*) for 19.47% of the total variation. Based on the geographical factor in influencing HBV evolution potentially, there is an obviously geographical distribution. For example, the overall codon usage pattern of HBV isolated from Philippines and South Korea is far from those of China and Indonesia, and the HBV isolated from Germany and Iran has a similar genetic diversity with that isolated from South Africa (Figure 1).

**Figure 2:**
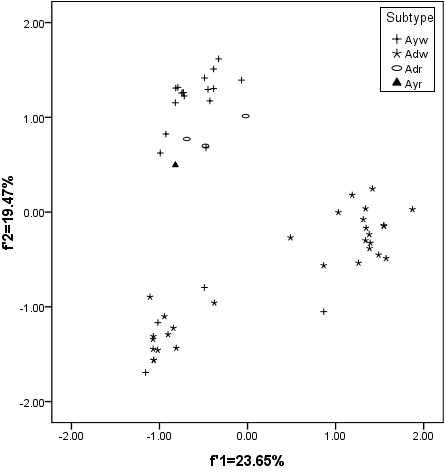
**The genetic characteristic of HBV based on the main four subtypes**.

Based on the subtypes of HBV, the plots for the subtype adw were generally divided into two groups, while the other three subtypes seem to have a similar genetic characteristic (Figure 2).

**Figure 3:**
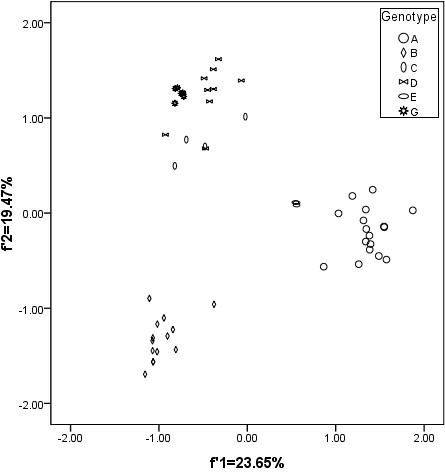
**The genetic characteristic of HBV based on different genotypes**.

It is worth noting that the plots for different HBV genotypes were generally separated from each other. Moreover, the genotypes A and B have an obviously different genetic characteristic with the rest, while genotypes C, D and G appear to have a relationship of evolution (Figure 3).

These results indicated that the geographic distribution might be a limited factor to effect the codon usage of the whole HBV coding sequence, and the subtypes did not reflect the characteristic of HBV evolution to some degree. In this case, the codon usage variation might be one of factors to drive HBV evolution.

The effect of mutation pressure on codon usage of HBV

**Figure 4:**
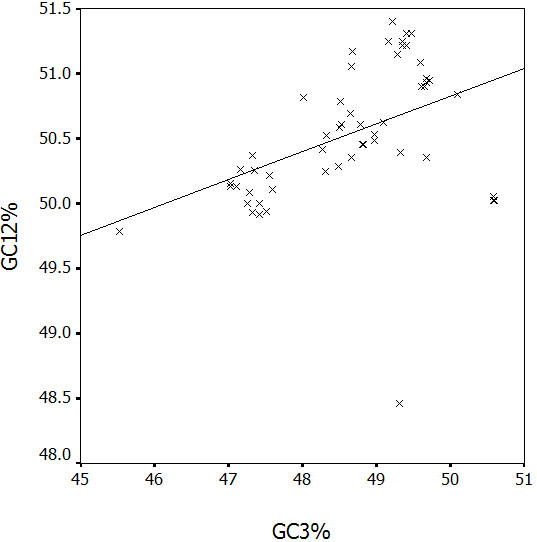
**Correlation between GC content at first and second codon positions (GC_2_%) with that at synonymous third codon positions (GC_3_%)**.

To analyze if the evolution of HBV is shaped by mutation pressure from virus itself or by translation selection from host, G+C content at the first and second codon positions (GC_12_%) was compared with that at synonymous third codon positions (GC_3_%) (Figure 4).

**Figure 5:**
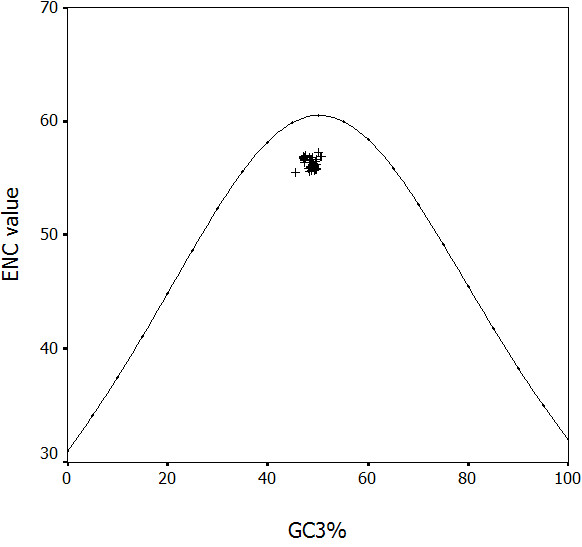
**Distribution of the codon usage index, ENC, and GC content at synonymous third codon positions (GC_3_%)**. The curve shows the expected codon usage of GC compositional constraints alone account for codon usage bias.

A highly significant correlation was observed (*r *= 0.432, P < 0.01), implying that mutation pressure from base composition of HBV is a main factor in shaping genetic diversity of this virus, since the effects are present at all codon positions. In addition, the ENC values were calculated for each strain and the plot was made by ENC value against GC_3_% (Figure 5).

The Figureure 5 represented that the plots of HBV aggregated below the expected curve, suggesting other selections take part in the process of HBV evolution.

### Comparative analysis of the RSCU values between HBV and human cell

There is a resemblance of synonymous codons usage pattern between this virus and human cell, for example, the similar synonymous codon usage pattern includes all synonymous codons for Phe, Ile, Val, Ser, Ala, Tyr, His, Lys, Asp, Cys and Gly (Table 1). This may be explained that the codon usage of HBV adapting to its host under translation selection could result in the multiplication of progeny virus. This phenomenon possibly implies that the resemblance of codon usage is favorable for HBV replication in human cells. But if compared with the under-represented codons in human cells, CCG for Pro, ACG for Thr, CAA for Gln and CUA for Leu in HBV are highly used (Table 1). The result suggested that these codons could influence the translational rate of the context flanking them, resulting in the viral product correct fold.

## Discussion

The ENC values calculated for HBV indicated that although a significantly lower bias of codon usage exists in HBV, the codon usage is not mainly affected by mutation pressure. As for some viruses, previous study reported that the major factor in shaping codon usage patterns appears to be mutation pressure rather than natural selection [19,21,24,35]. However, the comparison of the synonymous codon usage between HBV and human cells suggested that the interaction of mutation pressure with translation selection exists in the process of HBV evolution, although ENC values for the whole HBV coding sequence to represent mutation pressure is one of the factors in influencing codon usage pattern. This characteristic of HBV confers adaptive advantages which result in a highly efficient dissemination of the virus through different ways of transmission.

The pattern of codon usage is a genetic characteristic of various organisms in Previous study [19,20,27,31,32,35,36]. Because C%, U%, U_3_% and C_3_% play roles in the formation of the different optimal codons with any nucleotide-ended, the codon usage pattern of HBV is likely influenced by composition constraints. The codon usage pattern of PV is mostly coincident with that of its host, while the codon usage pattern of HBV is antagonistic to that of its host [37,38]. The codon usage pattern of HBV is a mixture of the two types of codon usage. The coincident portion of codon usage pattern for HBV enables the corresponding amino acids to be translated rapidly, the other antagonistic portion of codon usage pattern likely enable viral proteins to be folded properly, although the translation efficiency of the corresponding amino acids is decreased. Latent genes in Epstein-Barr virus deoptimize codon usage in order to evade competition for host protein translation [28] and attenuation of PV activity was performed by rare codon pairs inducing poor translation for sequences of viral proteins [27]. These results suggested that disfavored codons coding for amino acids may not be a deleterious factor for viruses to adapt to its host cells.

According to the data of codon usage pattern of HBV isolated from different countries, the geographic factor fails to influence the formation of codon usage pattern of HBV. After all, with development of international communication and highly efficient dissemination of HBV through various approaches of transmission, the affection of geographic factor seems to be weak on the limitation of HBV distribution in different countries. It is interesting that the main four subtypes of HBV have no significant difference in genetic characteristic shaped by different human races. This result might suggested that translation selection from human is not a single factor to shape the overall codon usage pattern of this virus and mutation pressure from HBV itself is a main force to drive HBV evolution. Genotyping of HBV is of high interest because there is increasing evidence that HBV genotypes may be associated with HBeAg sero-conversion rates, mutation occurring in the procure and core promoter region, severity of liver disease and treatment response [15,16,39,40]. There is a significant difference of the overall codon usage pattern of HBV between genotypes A, B, E and C, D, G. HBV genotypes and subgenotypes have been associated with differences in clinical and virological characteristics, showing that they may play a role in the virus-host relationship [41]. It has been shown that genotypes C and D are associated with more serious liver injuries and with a higher incidence of HCC than genotypes A and B [42-44]. In addition, genotype C and D have a much lower rate in response to interferon therapy than those infected with A or B genotypes [40,45]. Moreover, subtle differences in frequency and type of lamivudine resistant variants occur in genotype A and D infectious [15]. An evolutionary approach to HBV infection, based on the principles of natural selection, may offer explanation for how modes of transmission may favor some genotypes and subgenotypes over others and influence HBV virulence.

The genetic diversity and codon usage patterns we proposed here are helpful to understand the processes of HBV evolution, especially the roles played by translation selection from host and mutation pressure from virus. Additionally, such information might benefit to understand the roles of geographic and subtype factors in influencing the process of HBV evolution.

## Materials and methods

### Sequence data

The 58 complete RNA sequences of HBV were downloaded from the National Center for Biotechnology Information (NCBI) http://www.ncbi.nlm.nih.gov/Genbank/ and detailed information about the viruses were listed in Table 3

**Table 3:** The information of HBV strains in this study

**No**.	**Accession No**.	*f'1*^a^	*f'2*^a^	ENC value
1	AF405706	-0.79	1.32	56.41
2	X04615	-0.82	0.50	55.88
3	AB033554	-1.11	-0.90	55.78
4	AY741798	-0.82	1.31	56.17
5	AY741797	-0.82	1.15	55.82
6	AY741796	-0.72	1.23	56.62
7	AY741795	-0.75	1.26	56.59
8	AY741794	-0.73	1.26	56.61
9	AF100309	-1.02	-1.17	55.92
10	M57663	0.87	-1.05	55.48
11	AF100308	-1.16	-1.69	55.70
12	U87747	-0.38	-0.96	57.29
13	U87746	0.49	-0.27	55.71
14	AY123041	-0.69	0.77	55.94
15	AF068756	-0.48	0.70	56.39
16	AF282918	-0.84	-1.22	55.98
17	U95551	-0.99	0.62	56.36
18	GQ872210	-0.02	1.01	56.07
19	GQ161818	0.54	0.11	56.88
20	GQ161805	0.56	0.08	56.87
21	GQ161799	0.56	0.11	56.88
22	AY796032	-0.49	1.42	56.08
23	AY796031	-0.43	1.17	56.08
24	AY796030	-0.47	0.68	56.67
25	AF282917	-1.07	-1.45	55.70
26	AY233296	-0.07	1.39	55.62
27	AY23329	-0.38	1.30	56.04
28	AY233294	-0.33	1.62	55.95
29	AY233293	-0.39	1.51	55.92
30	AY233291	-0.45	1.29	55.95
31	AY233290	1.42	0.25	56.75
32	AY233289	1.57	-0.49	56.66
33	AY233288	1.39	-0.33	56.84
34	AY233287	1.55	-0.14	56.82
35	AY233286	1.03	0.00	56.78
36	AY233285	1.26	-0.54	56.52
37	AY233284	1.38	-0.24	56.78
38	AY233283	1.49	-0.45	56.54
39	AY233282	1.35	-0.17	56.73
40	AY233281	1.31	-0.08	56.95
41	AY233280	1.19	0.18	56.82
42	AY233279	1.34	0.04	56.90
43	AY233278	0.86	-0.56	56.37
44	AY233277	1.55	-0.15	56.88
45	AY233276	1.38	-0.38	56.83
46	AY233275	1.87	0.03	56.79
47	AY233274	1.34	-0.30	56.60
48	AY233273	-0.49	-0.80	56.45
49	DQ448628	-1.07	-1.31	55.84
50	DQ448627	-1.07	-1.56	55.84
51	DQ448625	-1.07	-1.56	55.68
52	DQ448623	-1.07	-1.34	55.76
53	DQ448622	-0.81	-1.44	55.90
54	DQ448621	-0.94	-1.10	56.24
55	DQ448620	-1.02	-1.46	55.77
56	DQ448620	-0.90	-1.29	56.01
57	AY373432	-0.73	1.26	56.61
58	AY373430	-0.93	0.82	55.86

^a ^*f'1 *and *f'2*, respectively, were calculated by PCA method.

Each general nucleotide composition (U%, A%, C% and G%) and each nucleotide composition in the third site of codon (U_3_%, A_3_%, C_3_% and G_3_%) in HBV coding sequence were calculated by biosoftware DNAStar 7.0 for windows.

### The calculation of the relative synonymous codon usage (RSCU)

The relative synonymous codon usage (RSCU) values for the whole 58 coding sequence of HBV were calculated as previously described [46]. RSCU values do not depend on the factors of amino acid composition and the size of the coding sequence, because the two factors can be eliminated in the process of calculation. When RSCU value is equal to 1.0, it means that this codon is chosen equally and randomly. The RSCU value for a synonymous codon more than 1.0 or less than 1.0 indicates the more frequency or less frequency, respectively. The synonymous codons with RSCU more than 1.6 were thought to be over-represented, while the synonymous codons with RSCU less than 0.6 were regarded as under-represented [47].

### Analysis of codon usage bias

The 'effective number of codons' (ENC), the useful estimator of absolute codon usage bias, was a measure quantifying the codon usage bias of the whole coding sequence of HBV. The ENC value ranges from 20 (when only one synonymous codon is chosen by the corresponding amino acid) to 61 (when all synonymous codons are used equally) [48]. In this study, this measure was used to evaluate the degree of codon usage bias of coding sequences for HBV.

### Principal component analysis

Principal component analysis (PCA), which was a commonly used multivariate statistical method [24], was carried out to analyze the major trend in codon usage pattern among different strains of HBV. PCA involves a mathematical procedure that transforms some correlated variable (RSCU values) into a smaller number of uncorrelated variables called principal components. Each strain was represented as a 59 dimensional vector, and each dimension corresponded to the RSCU value of each sense codon, which only included several synonymous codons for a particular amino acid, excluding the codon of AUG, UGG and three stop codons.

### Correlation analysis

The relationship between each general nucleotide composition (U%, A%, C% and G%) and each nucleotide composition in the third site of codon (U_3_%, A_3_%, C_3_% and G_3_%) in HBV coding sequence and the relationship between U_3_%, A_3_%, C_3_%, G_3_% and the coodn usage pattern of HBV were evaluated by the Pearson's rank.

All statistical processes were carried out by statistical software SPSS11.5 for windows.

## Author details

Experimental Center of Medicine, Lanzhou General Hospital, Lanzhou Military Area Command; Key lab of Stem cells and Gene Drugs of Gansu Province, Lanzhou 730000, China

## Competing interests

The authors declare that they have no competing interests.

## Authors' contributions

RMM and HL carried out the molecular genetic studies, participated in the sequence alignment and drafted the manuscript., MLW and FXZ participated in the sequence alignment. SDZ, GL and YW participated in the design of the study and performed the statistical analysis. XQH conceived of the study, and participated in its design and coordination and helped to draft the manuscript. All authors read and approved the final manuscript.
